# CNN-transformer-based model explained by SHAP and multi-head attention weights for time series forecasting

**DOI:** 10.1038/s41598-026-63013-0

**Published:** 2026-07-25

**Authors:** Stefano Frizzo Stefenon, João Pedro Matos-Carvalho, Valderi Reis Quietinho Leithardt, Kin-Choong Yow

**Affiliations:** 1 Instituto Superior de Engenharia de Lisboa, Instituto Politécnico de Lisboa, Rua Conselheiro Emídio Navarro 1, 1959-007 Lisboa, Portugal; 2 Faculty of Engineering and Applied Sciences, University of Regina, 3737 Wascana Parkway, Regina, Saskatchewan S4S 0A2 Canada; 3 LASIGE, Faculdade de Ciências, Universidade de Lisboa, 1749-016 Lisboa, Portugal; 4 Center of Technology and Systems (CTS-UNINOVA), Associated Laboratory of Intelligent Systems (LASI), 2829-516 Lisbon, Portugal; 5 Instituto Universitário de Lisboa (ISCTE-IUL), ISTAR, Avenida das Forças Armadas, 1649-026 Lisboa, Portugal

**Keywords:** Attention mechanism, Convolutions, Deep learning, Explainable artificial intelligence, Time series forecasting, Engineering, Mathematics and computing

## Abstract

Convolutional Neural Networks (CNNs) and transformer architectures offer strengths for modeling temporal data: CNNs excel at capturing local patterns and translational invariances, while transformers effectively model long-range dependencies via self-attention. This paper proposes a hybrid architecture integrating convolutional feature extraction with a multi-head attention backbone to enhance multivariate time series forecasting. The CNN module first applies a hierarchy of one-dimensional convolutional layers to distill salient local patterns from raw input sequences, reducing noise and dimensionality. The resulting feature maps are then fed into the prediction model, which applies multi-head attention to capture both short- and long-term dependencies and to weigh relevant covariates adaptively. We evaluate the proposed CNN-transformer-based model on a hydroelectric natural flow time series dataset. Experimental results demonstrate that the proposed model outperforms well-established deep learning models, with a mean absolute percentage error of up to 2.2%. The explainability of the model is obtained by a proposed SHapley Additive exPlanations (SHAP) and Multi-Head Attention Weights (MHAW). Our novel architecture, named CNN-Transformer-SHAP-MHAW, is promising for applications requiring high-fidelity and multivariate time series forecasts.

## Introduction

Time series forecasting is a fundamental problem in many fields, with applications ranging from energy systems and finance to network traffic analysis and cyber-physical infrastructures^1^. The task involves learning complex temporal dependencies from sequential data, which are often nonlinear, nonstationary, and influenced by multivariate interactions^2^. Despite significant progress, developing models that effectively capture both short-term dynamics and long-term dependencies while maintaining interpretability remains an open challenge.

Recent advances in deep learning have led to the development of architectures capable of modeling complex temporal data with high performance. Convolutional Neural Networks (CNNs) have demonstrated promising performance in extracting localized temporal features through hierarchical filtering, enabling the efficient representation of short-term patterns, such as trends and seasonal variations^3^. In parallel, Transformer-based architectures, driven by self-attention mechanisms, have redefined sequence modeling, enabling the direct modeling of long-range dependencies without relying on recurrent structures. These models offer improved scalability and parallelization, but may have limitations in capturing refined local temporal structures when applied directly to raw sequences^4^.

Hybrid architectures that combine convolutional and attention-based mechanisms have emerged as a promising direction for overcoming these limitations^5^. By integrating complementary inductive biases, such models aim to jointly exploit local feature extraction and global context modeling. Among attention-based approaches, transformer-based models have demonstrated strong performance in multivariate time series forecasting due to their ability to incorporate heterogeneous inputs and dynamically select relevant temporal features^6^. However, their effectiveness can be further enhanced by improving the quality of input representations through structured feature extraction mechanisms.

Beyond predictive performance, interpretability has become a critical requirement in modern machine learning systems^7^. Many deep learning models operate as black boxes, limiting their applicability in domains where transparency and reliability are essential. EXplainable Artificial Intelligence (XAI) techniques, such as SHapley Additive exPlanations (SHAP), provide post-hoc explanations by attributing contributions to the model’s input features^8^. However, existing approaches often fail to reconcile feature attribution with the internal decision processes of attention-based models, leading to fragmented or incomplete interpretations^9^.

Since different attention heads can capture distinct types of dependencies, such as short-term trends, seasonal behavior, or long-range relationships, Multi-Head Attention Weights (MHAW) offer a comprehensive interpretation of how the model processes sequential information. This intrinsic interpretability makes attention-based explanations particularly suitable for time series forecasting, enabling researchers to assess model behavior without requiring additional post hoc explanation methods^10^.

Based on the need to improve prediction and interpretability, this paper presents a new hybrid architecture, called CNN-Transformer-SHAP-MHAW, designed to address both predictive performance and interpretability in multivariate time series forecasting. The proposed approach integrates causal one-dimensional convolutional layers for extracting structured local features with a prediction architecture for modeling global temporal dependencies. To enhance interpretability, we propose a unified framework that combines SHAP-based feature attribution with MHAW, producing an influence representation that captures both the importance and contextual relevance of the input time steps.

Our proposed CNN-Transformer-SHAP-MHAW model has the following contributions:

- The hybrid structure combines CNN and attention outputs for enhanced feature extraction, ensuring that the most important features are used for signal prediction.
- Multi-head attention learns temporal relationships, capturing long-range dependencies, helping in the identification of relationships between distant time steps.
- Our model uses causal padding in convolutional layers to ensure predictions only depend on past/current inputs (no data leakage from future values).
- By merging attention weights with SHAP values, this approach creates a more interpretable and trustworthy influence map that reveals both what the model focuses on and why, enhancing explainability to support better decision-making.

The remainder of this paper is organized as follows: Section Related works presents a discussion of related works on CNNs, transformer-based architectures, attention mechanisms, and explainable artificial intelligence applied to time series forecasting. Section CNN-Transformer-SHAP-MHAW describes the proposed CNN-Transformer-SHAP-MHAW architecture, including the causal convolutional layers, multi-head self-attention mechanism, Bayesian optimization strategy, and the proposed explainability framework based on SHAP and attention weights. Section Results and discussion presents the experimental results, comparative analyses, statistical assessment, and explainable forecasting results. Finally, Section Conclusion concludes the paper and discusses future research directions.

## Related works

The literature on time series forecasting has evolved from traditional regression-based methods, which rely heavily on large volumes of historical and exogenous data but struggle with nonlinear and multivariate dynamics, to advanced deep learning architectures capable of modeling complex temporal dependencies^11^. Recurrent models have improved sequential learning but still have limitations in capturing long-term dependencies and in scalability, while CNNs have improved the extraction of local features through hierarchical representations of temporal patterns. More recently, hybrid models that combine convolutional structures with attention mechanisms have demonstrated superior performance by jointly leveraging local and global information^12^.

A combination of models that has been successfully applied for time series forecasting is the CNN with Long Short-Term Memory (LSTM) using the attention mechanism^13^, also called attention-based CNN-LSTM^14^. CNNs automatically extract hierarchical local patterns and temporal dependencies through convolutional filters, which reduces the need for manual feature engineering^15^. By sliding kernels across the input sequence, CNNs efficiently capture multi-scale trends, seasonality, and cyclical patterns while leveraging parameter sharing to reduce computational complexity. Their translation invariance allows them to recognize recurring motifs regardless of position, and their parallel processing capability accelerates training^16^.

The attention mechanism dynamically weighs the influence of historical observations, enabling the model to focus on relevant time steps while ignoring noise^17^. This adaptability enhances the capture of long-range dependencies, which is essential in sequences where distant past events influence future values^18^. Nevertheless, despite the integration of attention mechanisms, regression-based models remain limited in their ability to capture long-term trends and model complex nonlinear interactions among variables^19^, as their performance is inherently constrained by a strong reliance on historical data^20^. For these reasons, several authors have proposed variations on these models, and some of them will be explained here.

Aslam et al.^21^ proved that the two-stage attention mechanisms over LSTM networks have demonstrated superior performance in capturing complex temporal dependencies and emphasizing critical input features such as solar radiation, temperature, and humidity. The integration of Bayesian optimization for hyperparameter tuning has led to further improvements in prediction accuracy. Comparative evaluations with existing models, including CNN-LSTM, LSTM-attention, and other single attention frameworks, have shown that these advanced models offer enhanced forecasting skill and lower error metrics.

In arid regions such as Hail, Saudi Arabia, where climatic conditions are extreme and variable, Deep Learning (DL) architectures have shown promising capabilities for one-day-ahead irradiation forecasting. Models such as LSTM, bidirectional LSTM, Gated Recurrent Unit (GRU), bidirectional GRU, and their hybrid combinations, such as CNN-LSTM and CNN-BiLSTM, have been evaluated by Boubaker et al.^22^ using a 20-year daily irradiation dataset. Based on these analyses, they were able to obtain a correlation coefficient of up to 96%. Their findings highlight the potential of a hybrid DL model applied for enhancing solar resource forecasting in data-constrained or meteorologically harsh environments.

According to^23^, the CNN with Wavelet Neural Networks (WNN) and Masked Multi-Head Attention (MMHA) is a promising solution by integrating spatial feature extraction through CNNs, multi-scale frequency analysis via wavelet neural networks WNN, and temporal sequence modeling with MMHA. This hybrid architecture allows for a comprehensive understanding of irradiance behavior under diverse climatic conditions. Their model achieved a 79% reduction in Mean Absolute Percentage Error (MAPE), indicating improved prediction accuracy. The results also showed better generalization across varying weather conditions, with consistent performance in capturing both short-term fluctuations and long-term patterns in irradiance data.

XAI is an analysis trend that is being explored to improve the interpretability of model results^24^. In time series contexts, SHAP can help provide explanations for model predictions, helping to build trust and transparency^25^. By fairly distributing the contribution of each feature based on cooperative game theory (Shapley values), SHAP offers both global insights into feature importance and local explanations for individual predictions^26^. SHAP’s additive nature allows interpretation of how each feature influences the outcome, which is especially valuable for model validation^27^. By assigning an influence value to each feature at each time step, SHAP enables the interpretation of complex models, highlighting which inputs most significantly drive forecasts^28^.

Transformer-based forecasting models have been extended by incorporating generative diffusion models to improve long sequence time series forecasting. The Diffinformer architecture combines a conditional diffusion model with the ProbSparse self-attention and sequence distillation mechanisms of Informer, integrating the generated representations into the decoder to better capture long-range temporal dependencies. By leveraging the generative capability of diffusion models, this approach enhances forecasting performance while preserving the computational efficiency of Informer. Experimental evaluations on multiple large-scale datasets demonstrated that Diffinformer achieved higher predictive accuracy than conventional Transformer-based forecasting methods, highlighting the potential of diffusion models for long-term time series prediction^29^.

The Temporal Fusion Transformer (TFT) has emerged as a promising approach for time series forecasting due to its ability to model complex temporal relationships and effectively handle heterogeneous inputs, leading to its increasing adoption^30^. By leveraging multi-head self-attention, TFT dynamically captures both short- and long-range dependencies, enabling improved accuracy in noisy and multivariate datasets^31^. In contrast to sequential processing strategies that often face limitations in retaining long-term contextual information, TFT provides a more flexible and expressive framework for learning intricate temporal dynamics.

Hyperparameter tuning demonstrates potential in adapting model hyperparameters to the underlying data distribution, enabling the identification of optimal configurations that enhance predictive robustness on the given problem^21^. This process is particularly relevant in complex time series scenarios, where interactions between hyperparameters such as learning rate, network depth, and regularization terms can substantially influence model performance.

The nonlinear behavior induced by time series variability introduces substantial challenges in forecasting tasks, as data often exhibit abrupt fluctuations, seasonal patterns, and stochastic noise components^32^. These characteristics limit the effectiveness of single model approaches and highlight the suitability of hybrid frameworks, which combine complementary learning mechanisms to better capture both deterministic trends and stochastic variations.

The integration of CNN for feature extraction enhances the model’s ability to automatically learn hierarchical representations from raw input data. By capturing local temporal patterns and invariant features, CNN-based components improve the extraction of meaningful structures, which can then be leveraged by subsequent modules to refine predictions. This combination of advanced feature extraction and optimized model configuration reinforces the effectiveness of modern forecasting architectures in handling complex, real-world time series^33^. A summary of related works on DL-based time series forecasting covered in this paper is presented in Table 1.

**Table 1:** Summary of related works on DL-based time series forecasting.

Reference	Method	Highlights
^13,14^	Attention-based CNN-LSTM	Combines CNN feature extraction with LSTM temporal learning and attention mechanisms to improve forecasting accuracy and temporal dependency modeling.
^15,16^	CNN for time series forecasting	CNNs to extract hierarchical temporal patterns, reduce manual feature engineering, capture multi-scale trends, and accelerate training.
^17,18^	Attention mechanisms	Attention dynamically weights historical observations, improving long-range dependency capture and reducing the influence of noisy observations.
^19,20^	Regression-based forecasting models	Regression approaches still face limitations in modeling nonlinear interactions and long-term dependencies due to their reliance on historical observations.
^21^	Two-stage attention LSTM with Bayesian optimization	Improved forecasting by emphasizing meteorological features and optimizing hyperparameters, outperforming CNN-LSTM and single-attention models.
^32^	Convolutional autoencoders with clustering	Hybrid framework processes sky images to capture cloud spatial patterns and improve forecasting.
^22^	LSTM, BiLSTM, GRU, CNN-LSTM, CNN-BiLSTM	Hybrid DL models achieved correlation coefficients up to 96% for one-day-ahead irradiation forecasting in harsh climatic environments.
^23^	CNN-WNN-MMHA	Combines CNN, wavelet neural networks, and masked multi-head attention for multi-scale irradiance forecasting, achieving a 79% reduction in MAPE.
^24–28^	SHAP-based XAI	SHAP improves interpretability by quantifying feature contributions for forecasting models.
^30,31^	TFT	TFT effectively captures short- and long-term dependencies through multi-head self-attention, improving forecasting in noisy multivariate datasets.
^21^	Hyperparameter tuning	Hyperparameter tuning improves forecasting robustness by adapting hyperparameters to the data distribution.
^32^	Hybrid forecasting frameworks	Hybrid architectures improve forecasting performance by combining complementary learning mechanisms to model nonlinear and stochastic behaviors.
^33^	CNN-based feature extraction	CNN modules improve representation learning by capturing invariant local temporal structures.

Based on these previous research findings, this paper proposes a model created from scratch, called CNN-Transformer-SHAP-MHAW, optimized by Bayesian optimization for hydroelectric natural inflow forecasting. The proposed model uses causal convolutional blocks in the encoder, leveraging CNNs’ efficiency and parallelism on local patterns.

## CNN-Transformer-SHAP-MHAW

The proposed model is a hybrid DL architecture designed for time series forecasting, combining the strengths of CNNs and transformer-based attention mechanisms. The model begins with causal convolutional layers to efficiently extract local temporal features while preserving the autoregressive structure of time series data (presented in subsection Causal convolutional layers). These representations are then processed by a multi-head self-attention mechanism, which captures long-range dependencies and temporal relationships across the input window (see subsection Multi-head self-attention).

The proposed architecture differs from the TFT by adopting a simplified transformer-based design that combines causal one-dimensional convolutional layers with a multi-head self-attention mechanism. While TFT includes specialized components such as variable selection networks, gated residual networks, and recurrent encoder-decoder modules to process heterogeneous inputs^34^, the proposed method focuses on the complementary strengths of convolution and self-attention. Specifically, the convolutional layers efficiently learn local temporal patterns and short-term dependencies, whereas the multi-head attention mechanism captures long-range temporal relationships across the input sequence. This design reduces architectural complexity while retaining the transformer’s capability to model global temporal dependencies, providing an effective framework for multivariate time series forecasting.

**Fig. 1:**
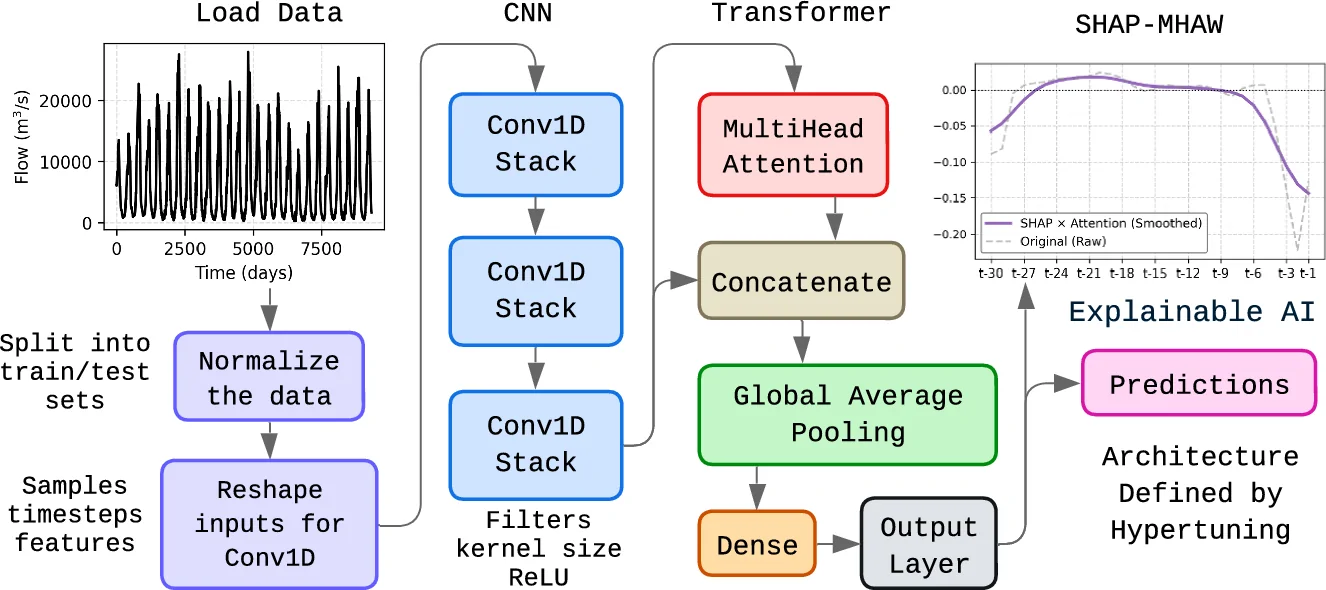
Summarized architecture of the proposed CNN-Transformer-SHAP-MHAW.

By fusing the outputs of both modules (feature extraction and prediction), the model leverages both short-term patterns and global context to make better predictions (see subsection Feature fusion and prediction). To ensure that the best hyperparameters are used, Bayesian optimization is employed; this method is explained in subsection Hyperparameter tuning by Bayesian optimization. The interpretability of the proposed SHAP-MHAW is explained in subsection Explainable results. Figure 1 presents the architecture of the proposed CNN-Transformer-SHAP-MHAW.

The input sequence is represented as $X \in \mathbb {R}^{w \times F}$, where *w* denotes the input window length and *F* is the number of input features. After the stack of causal convolutional layers, the resulting feature representation is denoted by $H_{\textrm{CNN}} \in \mathbb {R}^{w' \times d}$, where *d* is the number of learned feature channels. The query, key, and value matrices are computed as $Q, K, V \in \mathbb {R}^{w' \times d_k}$, producing the attention output $H_{\textrm{ATT}} \in \mathbb {R}^{w' \times d'}$. The feature fusion stage concatenates the CNN and attention representations, resulting in $H_{\textrm{fused}} \in \mathbb {R}^{w' \times (d+d')}$.

A global average pooling operation is then applied along the temporal dimension, yielding the feature vector $z \in \mathbb {R}^{d+d'}$, which is finally mapped by the dense output layer to the prediction vector $\hat{\textbf{y}} \in \mathbb {R}^{H}$, where *H* denotes the forecasting horizon. These definitions ensure that the tensor dimensions remain consistent throughout the CNN, attention, feature fusion, pooling, and prediction stages.

### Causal convolutional layers

To capture local temporal dynamics in the input time series, the model employs a stack of 1D causal convolutional layers (Conv1D). Given an input sequence $X \in \mathbb {R}^{w\times F}$, where *w* is the input window length and *F* is the number of input features, the first convolutional layer applies a 1D causal convolution with *f* filters and kernel size *k*, producing the feature representation $H^{(1)}\in \mathbb {R}^{w'\times d}$:

1 $$\begin{aligned} H^{(1)} = \text {ReLU}\left( \text {Conv1D}_{\text {causal}}^{(f,k)}(X)\right) , \end{aligned}$$

where $\textrm{Conv1D}^{(f,k)}_{\textrm{causal}}$ denotes a convolution with *f* filters of width *k* using causal padding. Let $\textbf{x}_{t-i}\in \mathbb {R}^{F}$ denote the multivariate input at time *t-i*, and $W_i\in \mathbb {R}^{d\times F}$ the corresponding convolution kernel. The output feature vector at time *t* is

2 $$\begin{aligned} \textbf{h}_t^{(1)} = \sigma \left( \sum _{i=0}^{k-1} W_i \textbf{x}_{t-i} + \textbf{b} \right) , \qquad \textbf{b}\in \mathbb {R}^{d}. \end{aligned}$$

where $\textbf{x}_{t-i}\in \mathbb {R}^{F}$ denotes the multivariate input feature vector at time *t-i*, $W_i\in \mathbb {R}^{d\times F}$ is the convolution kernel, $\textbf{b}\in \mathbb {R}^{d}$ is the bias vector, and $\textbf{h}_t^{(1)}\in \mathbb {R}^{d}$ is the output feature vector of the first convolutional layer at time *t*. Here, *d* denotes the number of output feature channels (filters).

A second identical causal convolution is then applied:

3 $$\begin{aligned} H^{(2)} = \text {ReLU}\left( \text {Conv1D}_{\text {causal}}^{(f,k)}(H^{(1)})\right) , \end{aligned}$$

resulting in deeper hierarchical features that encode increasingly abstract temporal patterns. By stacking causal convolutional layers with kernel size *k*, the model effectively captures dependencies across a short historical context^35^.

Each convolutional layer learns distinct filters that act as pattern detectors over local subsequences. The ReLU activation function introduces non-linearity, enabling the network to model complex, piecewise linear transformations of the input. Stacking multiple causal convolutional layers allows the network to model longer temporal dependencies while preserving an autoregressive structure^36^. This CNN block acts as a temporal feature extractor, transforming the raw time series into a higher-dimensional representation that emphasizes recent local structures, which is then passed to the attention mechanism for global reasoning.

### Multi-head self-attention

Following the convolutional layers, we incorporate a multi-head self-attention mechanism to model global temporal dependencies. Let $H \in \mathbb {R}^{w' \times d}$ be the output of the CNN stack. Self-attention computes attention scores between all time steps:

4 $$\begin{aligned} \text {Attention}(Q, K, V) = \text {softmax}\left( \frac{QK^\top }{\sqrt{d_k}}\right) V, \end{aligned}$$

where $H\in \mathbb {R}^{w'\times d}$ is the output of the CNN block, $Q,K,V\in \mathbb {R}^{w'\times d_k}$ are the query, key, and value matrices obtained through the linear projections

$$\begin{aligned} Q=HW_Q,\qquad K=HW_K,\qquad V=HW_V, \end{aligned}$$

with $W_Q,W_K,W_V\in \mathbb {R}^{d\times d_k}$ being learnable projection matrices^37^.

In Multi-Head Attention (MHA), this operation is performed in parallel over *h* different heads:

5 $$\begin{aligned} \text {MHA}(H) = \text {Concat}(\text {head}_1, \ldots , \text {head}_h)W^O, \end{aligned}$$

where each attention head has dimension $d_k$, the concatenated representation has dimension $w'\times (hd_k)$, and $W_O\in \mathbb {R}^{hd_k\times d'}$ projects the concatenated heads into the output representation $H_{\textrm{ATT}}\in \mathbb {R}^{w'\times d'}$^ 38^.

### Feature fusion and prediction

The CNN output $H_{\textrm{CNN}}\in \mathbb {R}^{w'\times d}$ and the attention output $H_{\textrm{ATT}}\in \mathbb {R}^{w'\times d'}$ are concatenated along the feature dimension to form the fused representation:

6 $$\begin{aligned} H_\text {fused} = \text {Concat}(H_\text {CNN}, H_\text {ATT}) \in \mathbb {R}^{w' \times (d + d')}. \end{aligned}$$

To aggregate temporal information while preserving the learned feature representation, a global average pooling operation is applied along the temporal dimension, resulting in the feature vector $z\in \mathbb {R}^{d+d'}$:

7 $$\begin{aligned} z = \frac{1}{w'} \sum _{t=1}^{w'} H_\text {fused}^{(t)} \in \mathbb {R}^{d + d'}. \end{aligned}$$

Finally, the pooled feature vector $z\in \mathbb {R}^{d+d'}$ is mapped by a dense output layer to the prediction vector $\hat{\textbf{y}}\in \mathbb {R}^{H}$, where *H* denotes the forecasting horizon:

8 $$\begin{aligned} \hat{\textbf{y}}=W_{\textrm{out}}z+\textbf{b}_{\textrm{out}}, \quad W_{\textrm{out}}\in \mathbb {R}^{H\times (d+d')}, \quad \textbf{b}_{\textrm{out}}\in \mathbb {R}^{H}, \quad \hat{\textbf{y}}\in \mathbb {R}^{H}. \end{aligned}$$

The model is trained using the mean squared error loss function ($\mathcal {L}$) over a training dataset $\{(x^{(i)}, y^{(i)})\}_{i=1}^{N}$:

9 $$\begin{aligned} L(\theta )= \frac{1}{N} \sum _{i=1}^{N} \left\| \textbf{y}^{(i)} - f_\theta (\textbf{x}^{(i)}) \right\| _2^2. \end{aligned}$$

The model’s architecture is promising for time series forecasting because it combines the strengths of convolutional and transformer-based models. The causal convolutional layers efficiently capture short-term temporal patterns and local dependencies with low computational cost, while ensuring that predictions respect the chronological order of data.

On top of this, the multi-head self-attention mechanism from the transformer framework enables the model to learn long-range dependencies and temporal relationships that may span across the entire input window, something CNNs alone struggle with. By concatenating both feature representations and using global pooling to summarize temporal dynamics, the model balances efficiency, making it well-suited for complex forecasting tasks where both local trends and global context are important.

### Hyperparameter tuning by Bayesian optimization

In this paper, Bayesian optimization is used for hyperparameter tuning, where we wish to solve the optimization problem

10 $$\begin{aligned} \textbf{x}^* \;=\;\arg \max _{\textbf{x}\in \mathcal {X}}\,f(\textbf{x}), \end{aligned}$$

where $\mathcal {X}\subset \mathbb {R}^d$ is the hyperparameter domain and *f* is expensive to evaluate^39^. We place a Gaussian process ($\mathcal{G}\mathcal{P}$) prior on *f*:

11 $$\begin{aligned} f(\textbf{x}) \;\sim \;\mathcal{G}\mathcal{P}\bigl (m(\textbf{x}),\,k(\textbf{x},\textbf{x}')\bigr ), \end{aligned}$$

where $m:\mathcal {X}\rightarrow \mathbb {R}$ is the mean function (often zero) and $k:\mathcal {X}\times \mathcal {X}\rightarrow \mathbb {R}$ is the covariance kernel (e.g. squared exponential)

12 $$\begin{aligned} k(\textbf{x},\textbf{x}') \;=\;\sigma _f^2\exp \!\Bigl (-\tfrac{1}{2}(\textbf{x}-\textbf{x}')^\top \Lambda ^{-1}(\textbf{x}-\textbf{x}')\Bigr ). \end{aligned}$$

Given observed data $\mathcal {D}_n=\{(\textbf{x}_i,y_i)\}_{i=1}^n$, where $y_i=f(\textbf{x}_i)+\varepsilon _i$ with $\varepsilon _i\sim \mathcal {N}(0,\sigma _n^2)$, the $\mathcal{G}\mathcal{P}$ posterior at a candidate $\textbf{x}$ is Gaussian:

13 $$\begin{aligned} \begin{aligned} \mu _n(\textbf{x})&= k(\textbf{x},X)\bigl [K + \sigma _n^2 I\bigr ]^{-1}\textbf{y},\\ \sigma _n^2(\textbf{x})&= k(\textbf{x},\textbf{x}) - k(\textbf{x},X)\bigl [K + \sigma _n^2 I\bigr ]^{-1}k(X,\textbf{x}), \end{aligned} \end{aligned}$$

where $$X=[\textbf{x}_1,\dots ,\textbf{x}_n]^\top$$, $$\textbf{y}=[y_1,\dots ,y_n]^\top$$, and $$K_{ij}=k(\textbf{x}_i,\textbf{x}_j)$$.

An acquisition function $\alpha _n(\textbf{x})$ balances exploration and exploitation^40^. Common choices include, where the Expected Improvement (EI) is:

14 $$\begin{aligned} \begin{aligned} \alpha _{\textrm{EI}}(\textbf{x}) \;=\;\mathbb {E}_{f\sim \mathcal {N}(\mu _n,\sigma _n^2)}\bigl [\max (f(\textbf{x}) - f^+,\;0)\bigr ] = \\ \sigma _n(\textbf{x})\bigl (u\,\Phi (u) + \varphi (u)\bigr ), \end{aligned} \end{aligned}$$

where

15 $$\begin{aligned} u = \frac{\mu _n(\textbf{x}) - f^+ - \xi }{\sigma _n(\textbf{x})}, \quad f^+ = \max _{i\le n} y_i, \end{aligned}$$

*Φ* and *φ* denote the standard normal cumulative distribution function and probability density function, and $\xi \ge 0$ is an optional exploration parameter^41^.

Bayesian optimization navigates expensive-to-evaluate hyperparameter spaces by modeling the unknown objective with a $\mathcal{G}\mathcal{P}$ surrogate, quantifying uncertainty to trade off exploration and exploitation via acquisition functions, and iteratively proposing new hyperparameter settings that maximize information gain or potential improvement^42^.

Table 2 presents the evaluation range of the hyperparameter analyzed in this paper. These values were considered since higher values of these hyperparameters resulted in a model that did not converge in some experiments.

**Table 2:** Evaluation hyperparameters range.

Hyperparameter	Minimum	Maximum	Step
CNN_layers	1	12	1
num_heads	2	5	1
filters	16	256	1
kernel_size	2	5	1

Bayesian optimization was performed using a validation subset corresponding to 20% of the chronological training partition. The validation RMSE was used as the objective function for hyperparameter selection, while the test set remained unseen during model selection and was used only for the final evaluation.

### Explainable results

To improve the interpretability of our model applied to univariate time series forecasting, two complementary explainability techniques were employed: SHAP with multi-head attention visualization. Algorithm 1 presents pseudo-code that depicts these descriptions.

**Algorithm 1:**
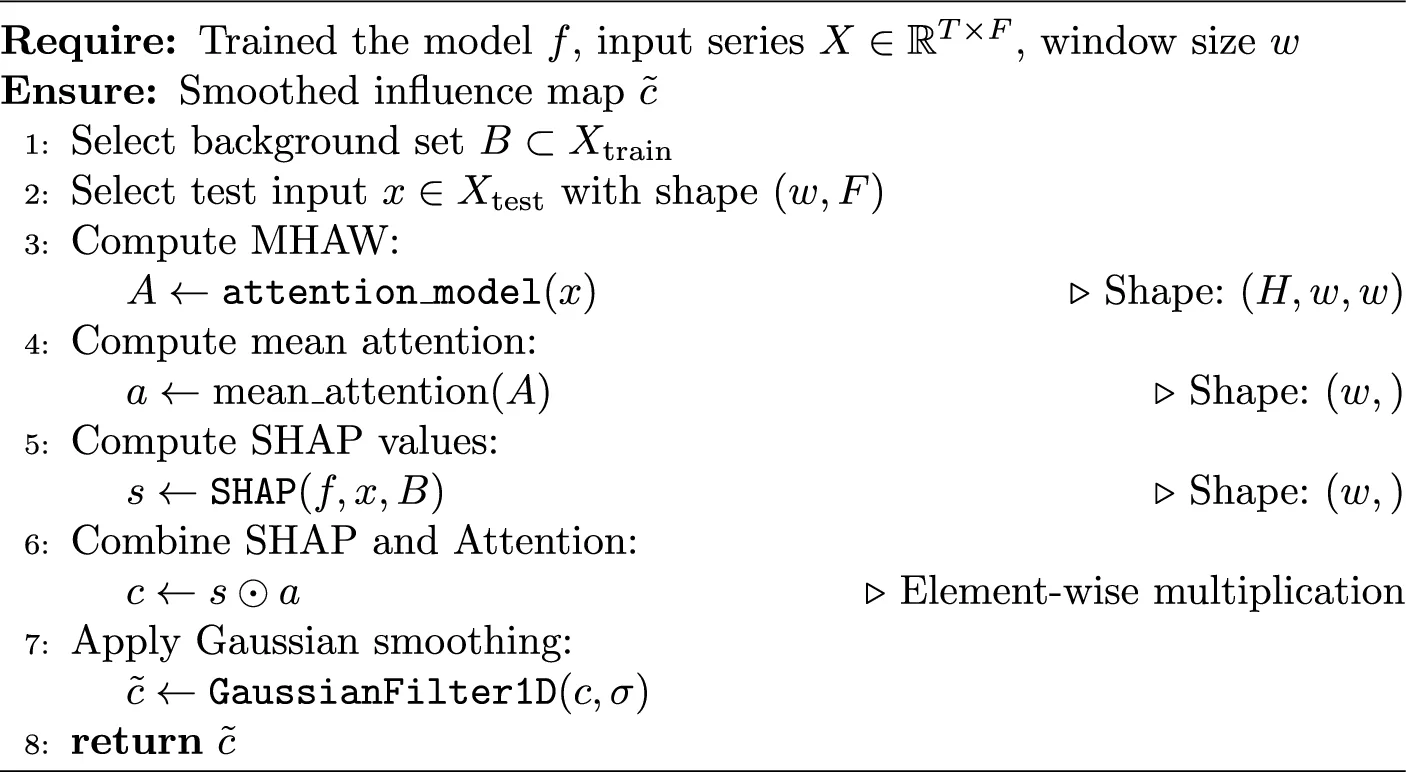
Explainability pipeline with SHAP-MHAW.

SHAP is used here to quantify the contribution of each time step within the input window to the model’s prediction. For each prediction, SHAP assigns an influence value to every lag. Considering that lag refers to a previous time step’s value of a time-dependent variable used as an input to predict future values. Positive SHAP values indicate that a given input step increased the predicted value, while negative values represent steps that pushed the forecast downward. The absolute magnitude of the SHAP values reflects the impact of each lag, regardless of direction^43^.

The combination of SHAP values and multi-head attention weights is motivated by their complementary interpretability capabilities. SHAP provides feature attribution by estimating the contribution of each input variable to the model prediction, whereas the attention weights indicate the temporal dependencies emphasized by the transformer-based attention mechanism during prediction. Consequently, SHAP explains *what* input features contribute most to the forecasting result, while the attention weights provide insight into *when* the model concentrates its attention across the input sequence. Their joint analysis therefore offers a more comprehensive interpretation of the learned forecasting behavior by integrating feature attribution with temporal relevance.

To synthesize both perspectives, the SHAP values were element-wise multiplied by the corresponding MHAW, resulting in a combined influence map (SHAP-MHAW). This map highlights input time steps that were both attended to and had an influence on the forecast. For future comparisons, the proposed CNN-Transformer-SHAP-MHAW is available for future analysis at: https://github.com/SFStefenon/CNN-Transformer-SHAP-MHAW.

## Results and discussion

This section presents the results and discussions of the application of the proposed CNN-Transformer-SHAP-MHAW model compared to well-established models.

### Experiment setup and dataset

The NVIDIA RTX 5000 Graphics Processing Unit (GPU), 16 GB of double data rate 6 synchronous dynamic random-access memory, was employed in this study to support computationally intensive tasks. The GPU was integrated into a Linux-based cluster and interfaced using compute unified device architecture to ensure compatibility with the DL and simulation frameworks utilized in this research.

**Fig. 2:**
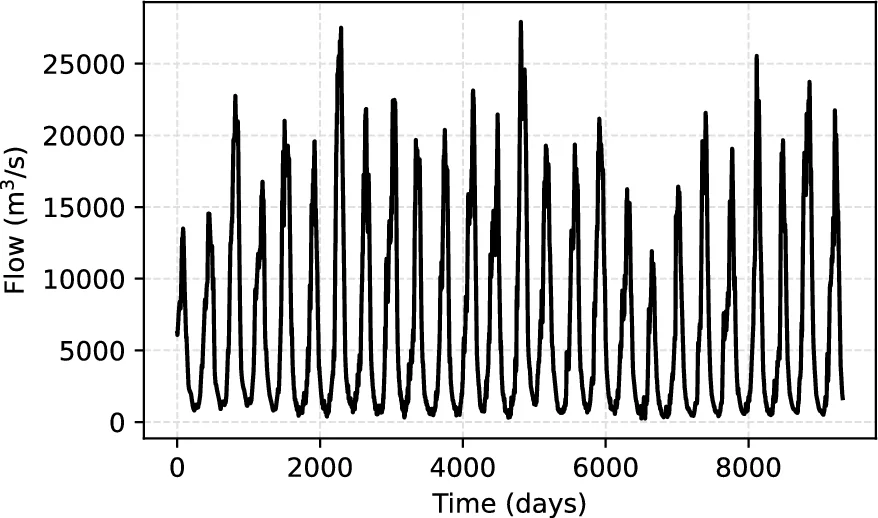
Original flow time series data.

The proposed CNN-Transformer-SHAP-MHAW was written using the Python language. For model assessment, the Root Mean Squared Error (RMSE), Mean Absolute Error (MAE), MAPE, and Mean Squared Logarithmic Error (MSLE)^44^ were considered. To evaluate the model, the historical flow record of a hydroelectric plant (Tucuruí) located in Brazil was used^12^. Figure 2 shows the original time series. This record covers a time window from January 2, 1998 to July 9, 2023, which is a daily record comprising 9,321 recorded values.

The forecasting task is formulated as a supervised sliding-window prediction problem. Given an input sequence of the previous *w* time steps, the model predicts the next *H* future values, where *H* denotes the forecasting horizon. Separate models are trained for each evaluated horizon (*H*=15, 30, and 60 time steps), using an input window equal to the forecasting horizon (*w=H*). Each training sample therefore consists of an input tensor of shape (*H*, *F*), where *F* is the number of input features, and a corresponding target sequence of length *H*.

The dataset is divided chronologically into 80% for training and 20% for testing to preserve the temporal ordering of the observations and prevent information leakage. During inference, the model directly predicts the complete forecasting horizon rather than recursively generating one-step-ahead forecasts. Bayesian optimization was performed using a validation subset corresponding to 20% of the chronological training partition, which was automatically created through the validation_split parameter during model training. The validation error (RMSE) was used as the optimization objective, while the test set remained completely unseen during hyperparameter selection and was used only for the final evaluation.

To perform a comparative analysis, we evaluate our algorithm against the Neural Basis Expansion Analysis with exogenous variables (NBEATSx)^45^, LSTM^46^, GRU^47^, DeepAR^48^, TFT^49^, informer^50^, Patch Time Series Transformer (PatchTST)^51^, Frequency Enhanced Decomposed transformer (FEDformer)^52^, Temporal Convolutional Network (TCN)^53^, and TimesNet^54^.

### Structural analysis

To improve the model’s predictive capacity, some structural modifications were evaluated. Table 3 shows the results of different structure variations (STV). The first change to the structure (M1) was through the use of just one convolutional layer (Conv1D), using batch normalization and dropout to stabilize training and reduce overfitting. The second change (M2) in the evaluated structure was the inclusion of the normalization layer to normalize inputs before the attention layer for better convergence.

The third structure proposal (M3) was to use a dense layer stacked, where hidden layers are added with dropout before the final prediction. The final modification evaluated (M4) used two convolutional layers (Conv1D), increasing the complexity of the convolutional structure.

**Table 3:** Results of different structural variations.

STV	RMSE	MAE	MAPE	MSLE
M1	2.43$\times 10^3$	2.09$\times 10^3$	7.10$\times 10^{-1}$	2.19
M2	1.46$\times 10^{7}$	1.41$\times 10^{7}$	4.34$\times 10^3$	7.00$\times 10^1$
M3	3.94$\times 10^3$	3.92$\times 10^3$	1.26	6.66$\times 10^{-1}$
M4	1.24$\times 10^2$	1.10$\times 10^2$	3.53$\times 10^{-2}$	1.54$\times 10^{-3}$

The normalization layer introduced in structure M2 resulted in substantially larger prediction errors than the remaining configurations. We verified that this behavior was not caused by an implementation error but by unstable optimization, as the normalization strategy impaired convergence under the evaluated architecture and hyperparameter configuration. Although M2 did not produce competitive forecasting performance, its inclusion highlights that not all architectural modifications improve model behavior, motivating the selection of the M4 configuration for the subsequent analyses.

The modification to the structure that most improved the model’s ability to make predictions was the use of more convolutional layers, which is why only this modification was used (structure M4, which is explained in Section Related works). Attempts to use hybrid pooling, teacher forcing, or dilated convolution were also unsuccessful and were therefore not implemented. Based on this discovery, the structure M4 is hypertuned to fine-tune its hyperparameters.

### Fine tuning

**Fig. 3:**
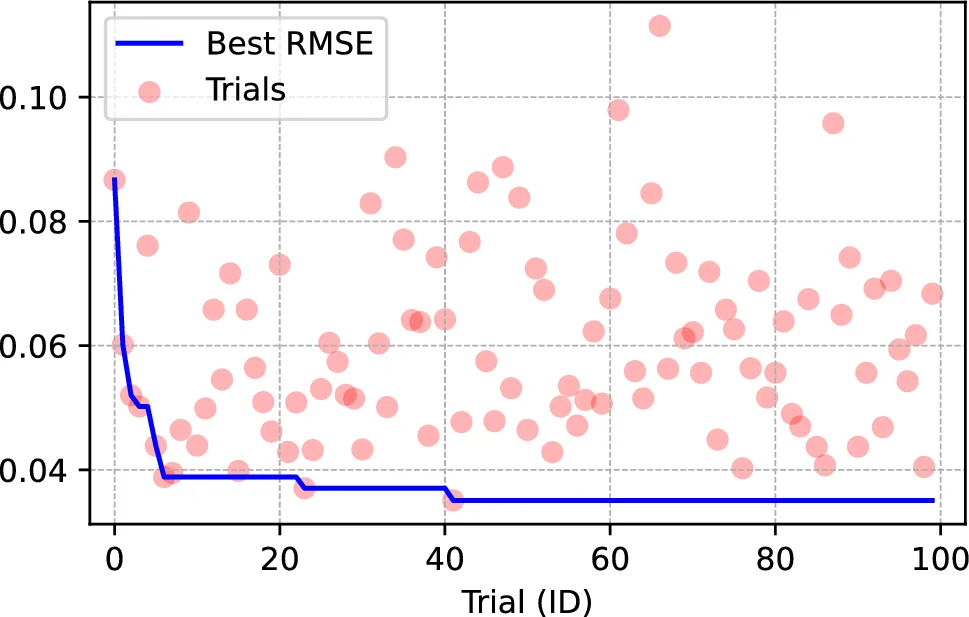
RMSE of the trial using Bayesian optimization for hyperparameter tuning.

The purpose of fine-tuning the model is to determine the optimal hyperparameters to be used in its structure, thus ensuring its best use. The Bayesian optimization hyperparameter tuning performs an interactive search to find the hyperparameters that minimize the loss function, which in this case is the RMSE. Figure 3 presents the loss (RMSE) of each trial in the experiments. The dots are the RMSE of each trial, and the blue line is the lowest loss seen by the Bayesian optimization method compared to earlier trials. A trial is a combination of hyperparameters considered by the Bayesian optimization method during hyperparameter tuning.

The results of the Bayesian optimization were 3 CNN layers, 4 heads for the attention mechanism, and 238 filters in the CNN using a kernel size of 4. Considering that all the hyperparameters were within the search space in the optimization process, the evaluation was adequate. Therefore, these values are used in our model.

**Fig. 4:**
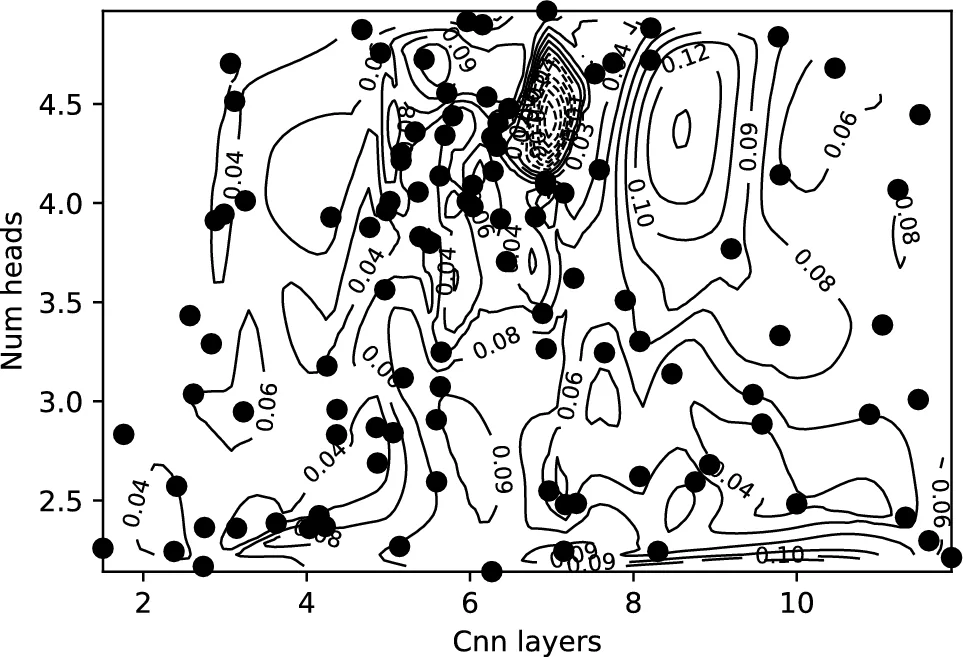
RMSE gradients of number of heads versus CNN layers.

Figure 4 shows the contour plot of RMSE values for a range of numbers of heads and CNN layers. The Results show that using 3 to 7 CNN layers, lower RMSE values are achieved, with a large variation in the number of heads, concerning the attention mechanism. The darker color points show the best results concerning the loss function, in this case, the lowest RMSE. The generation of the colormap of this plot involves calculating color gradients based on perceptual color space interpolation. The model tries to find a combination that reduces the loss function by considering a multi-criteria optimization of all the hyperparameters evaluated in the model.

**Fig. 5:**
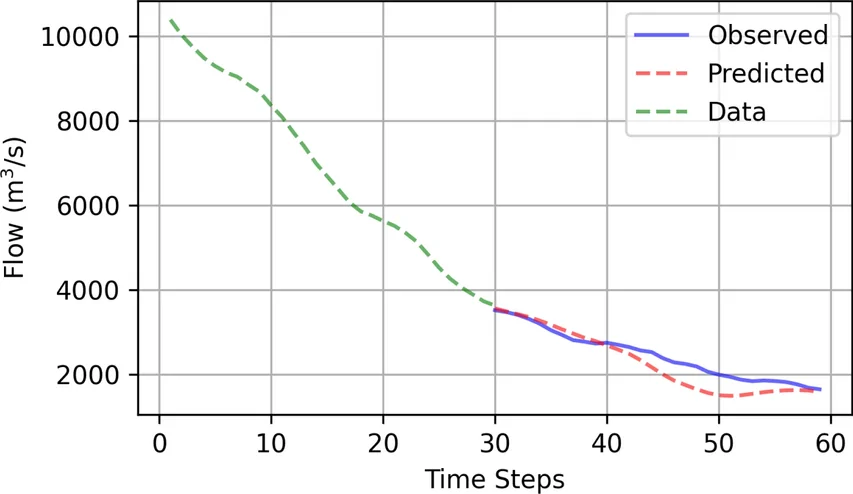
Original versus predicted time series by CNN-Transformer-SHAP-MHAW.

Once these hyperparameters have been defined, the model is retrained to be applied to the original data; Figure 5 presents an example of prediction comparison to the original signal. These values of hyperparameters are also used for the statistical assessment, which is presented in the next subsection.

### Comparative analysis

In this paper, we consider an input size equivalent to the forecasted horizon to train the model. Where the horizon refers to how far into the future you want to predict (time steps). The models were assessed using the RMSE, MAE, MAPE, and MSLE metrics, together with the computational execution time. The comparison includes recurrent architectures, transformer-based methods, convolutional approaches, and the proposed framework. Based on the optimal hyperparameter setup, Table 4 presents a comparative analysis (benchmarking) to compare the proposed CNN-transformer-based model (ours) to other well-established models for a horizon equal to 15 steps ahead.

**Table 4:** Comparative analysis of DL models for horizon 15.

Model	RMSE	MAE	MAPE	MSLE	Time (s)
NBEATSx	6.36$\times 10^2$	5.82$\times 10^2$	1.79$\times 10^{-1}$	3.30$\times 10^{-2}$	3.32$\times 10^{-1}$
LSTM	1.39$\times 10^3$	1.38$\times 10^3$	4.04$\times 10^{-1}$	1.18$\times 10^{-1}$	2.91$\times 10^{-1}$
GRU	5.68$\times 10^2$	4.51$\times 10^2$	1.24$\times 10^{-1}$	1.82$\times 10^{-2}$	**2.69**$\times 10^{-1}$
DeepAR	3.55$\times 10^3$	3.51$\times 10^3$	1.00	6.39$\times 10^1$	6.52$\times 10^{-1}$
TFT	4.85$\times 10^2$	4.05$\times 10^2$	1.24$\times 10^{-1}$	2.04$\times 10^{-2}$	2.03
Informer	3.54$\times 10^3$	3.51$\times 10^3$	9.99$\times 10^{-1}$	4.53$\times 10^1$	1.50
PatchTST	5.95$\times 10^2$	5.16$\times 10^2$	1.57$\times 10^{-1}$	2.76$\times 10^{-2}$	3.80$\times 10^{-1}$
FEDformer	2.89$\times 10^3$	2.84$\times 10^3$	8.49$\times 10^{-1}$	3.86$\times 10^{-1}$	2.73
TCN	1.69$\times 10^3$	1.45$\times 10^3$	4.28$\times 10^{-1}$	2.44$\times 10^{-1}$	3.98$\times 10^{-1}$
TimesNet	1.27$\times 10^3$	1.01$\times 10^3$	3.16$\times 10^{-1}$	1.03$\times 10^{-1}$	3.73
Ours	**9.28**$\times 10^1$	**7.37**$\times 10^1$	**2.20**$\times 10^{-2}$	**6.88**$\times 10^{-4}$	1.90$\times 10^1$

For the 15-step forecasting horizon, the proposed method achieved the best predictive performance among all evaluated DL models, obtaining the lowest RMSE, MAE, MAPE, and MSLE values. The proposed approach reached an RMSE of $9.28\times 10^1$ and an MAE of $7.37\times 10^1$, significantly outperforming transformer-based architectures such as TFT and PatchTST, as well as recurrent models including LSTM and GRU. The MAPE of $2.20\times 10^{-2}$ and the MSLE of $6.88\times 10^{-4}$ further demonstrate the robustness of the proposed model in capturing the temporal dynamics while maintaining very low relative and logarithmic errors.

Among the baseline models, TFT presented the second-best overall predictive accuracy, with an RMSE of $4.85\times 10^2$ and an MAE of $4.05\times 10^2$, followed by GRU and PatchTST. NBEATSx also showed competitive results, indicating that advanced neural forecasting architectures can effectively model short-term dependencies. In contrast, DeepAR and Informer exhibited substantially higher error values, suggesting limitations in learning the underlying temporal structure for this specific forecasting task. FEDformer, TCN, and TimesNet also produced higher errors compared with the best-performing methods.

Table 5 presents the comparative performance analysis of the evaluated DL models for the 30-step forecasting horizon. For 30 steps ahead, the proposed method achieved the best predictive performance among all evaluated approaches, obtaining the lowest values for RMSE, MAE, MAPE, and MSLE. Specifically, the proposed framework reached an RMSE of $2.01\times 10^2$ and an MAE of $1.76\times 10^2$, substantially outperforming all baseline models. The obtained MAPE of $7.40\times 10^{-2}$ and MSLE of $8.10\times 10^{-3}$ further demonstrate the ability of the proposed approach to preserve forecasting stability while minimizing both relative and logarithmic prediction errors.

**Table 5:** Comparative analysis of DL models for horizon 30.

Model	RMSE	MAE	MAPE	MSLE	Time (s)
NBEATSx	4.55$\times 10^2$	4.33$\times 10^2$	1.54$\times 10^{-1}$	2.23$\times 10^{-2}$	**2.37**$\times 10^{-1}$
LSTM	4.28$\times 10^3$	4.25$\times 10^3$	1.59	9.09$\times 10^{-1}$	2.88$\times 10^{-1}$
GRU	1.44$\times 10^3$	1.27$\times 10^3$	4.46$\times 10^{-1}$	1.47$\times 10^{-1}$	2.85$\times 10^{-1}$
DeepAR	3.01$\times 10^3$	2.92$\times 10^3$	1.00	6.08$\times 10^1$	5.35$\times 10^{-1}$
TFT	9.76$\times 10^2$	8.12$\times 10^2$	2.49$\times 10^{-1}$	1.19$\times 10^{-1}$	3.04
Informer	3.00$\times 10^3$	2.91$\times 10^3$	9.99$\times 10^{-1}$	4.44$\times 10^1$	1.40
PatchTST	4.47$\times 10^2$	3.78$\times 10^2$	1.50$\times 10^{-1}$	3.83$\times 10^{-2}$	4.28$\times 10^{-1}$
FEDformer	5.41$\times 10^3$	5.35$\times 10^3$	2.02	1.22	3.42
TCN	2.98$\times 10^3$	2.39$\times 10^3$	8.91$\times 10^{-1}$	6.21$\times 10^{-1}$	2.64$\times 10^{-1}$
TimesNet	1.99$\times 10^3$	1.67$\times 10^3$	6.04$\times 10^{-1}$	8.05$\times 10^{-1}$	8.38
Ours	**2.01**$\times 10^2$	**1.76**$\times 10^2$	**7.40**$\times 10^{-2}$	**8.10**$\times 10^{-3}$	2.09$\times 10^1$

Among the competing methods, PatchTST and NBEATSx achieved the best overall baseline performances. PatchTST obtained an RMSE of $4.47\times 10^2$ and an MAE of $3.78\times 10^2$, while NBEATSx presented competitive results with an RMSE of $4.55\times 10^2$ and an MAE of $4.33\times 10^2$. TFT also produced relatively consistent results compared with the remaining architectures, although with significantly larger errors than the proposed method. In contrast, recurrent models such as LSTM and GRU showed a noticeable degradation in performance as the forecasting horizon increased, indicating greater difficulty in modeling longer temporal dependencies. DeepAR, Informer, FEDformer, TCN, and TimesNet also exhibited considerably larger forecasting errors, especially in terms of RMSE and MAE.

Table 6 presents the comparative performance analysis of the evaluated DL models for the 60-step forecasting horizon. For this forecasting horizon, the proposed model achieved the best overall predictive performance, obtaining the lowest RMSE, MAE, and MAPE values among all evaluated models. Specifically, the proposed method reached an RMSE of $2.92\times 10^2$ and an MAE of $2.23\times 10^2$, demonstrating a substantial improvement compared with all baseline approaches.

**Table 6:** Comparative analysis of DL models for horizon 60.

Model	RMSE	MAE	MAPE	MSLE	Time (s)
NBEATSx	5.02$\times 10^2$	4.31$\times 10^2$	1.97$\times 10^{-1}$	**3.83**$\times 10^{-2}$	3.42$\times 10^{-1}$
LSTM	1.22$\times 10^4$	1.07$\times 10^4$	6.62	3.62	**3.32**$\times 10^{-1}$
GRU	3.05$\times 10^3$	2.86$\times 10^3$	1.49	8.40$\times 10^{-1}$	3.59$\times 10^{-1}$
DeepAR	2.36$\times 10^3$	2.18$\times 10^3$	1.00	5.68$\times 10^1$	1.23
TFT	2.18$\times 10^3$	2.01$\times 10^3$	9.18$\times 10^{-1}$	6.47	5.47
Informer	2.36$\times 10^3$	2.18$\times 10^3$	9.98$\times 10^{-1}$	3.83$\times 10^1$	3.75
PatchTST	1.76$\times 10^3$	1.53$\times 10^3$	9.39$\times 10^{-1}$	1.35	5.85$\times 10^{-1}$
FEDformer	1.11$\times 10^4$	1.11$\times 10^4$	6.12	3.72	1.84$\times 10^1$
TCN	6.00$\times 10^3$	5.03$\times 10^3$	2.84	1.77	4.24$\times 10^{-1}$
TimesNet	4.20$\times 10^3$	3.34$\times 10^3$	1.64	8.97$\times 10^{-1}$	1.66$\times 10^1$
Ours	**2.92**$\times 10^2$	**2.23**$\times 10^2$	**1.37**$\times 10^{-1}$	3.89$\times 10^{-2}$	2.16$\times 10^1$

The proposed model also obtained the lowest MAPE value of $1.37\times 10^{-1}$, indicating superior relative forecasting accuracy. Although NBEATSx achieved a slightly lower MSLE value of $3.83\times 10^{-2}$ compared with the proposed method value of $3.89\times 10^{-2}$, the difference is minimal, showing that both methods maintained stable logarithmic prediction behavior.

Regarding computational efficiency, LSTM achieved the lowest execution time with $3.32\times 10^{-1}$ s, followed closely by NBEATSx and GRU. The proposed method required the highest computational time of $2.16\times 10^1$ s, which is expected due to the increased complexity of the framework. Nevertheless, the significant reduction in forecasting errors demonstrates that the additional computational cost leads to a substantial gain in predictive accuracy and robustness. The results indicate that the proposed framework maintains strong generalization capability and superior forecasting stability even for longer prediction horizons.

### Statistical assessment

After hyperparameter tuning, we select the values of each hyperparameter, then we use these values to compute experiments (runs) with initialization of the random weights. We performed the analysis on 50 runs. Each run is computed by training the model up to 100 epochs and evaluating it, using 80% of the data for training and 20% for testing.

Table 7 shows the results of the statistical analysis of the RMSE, MAE, MAPE, and MSLE for 50 runs, where the mean, median, standard deviation, first quartile, third quartile, IQR, Skewness, and Kurtosis of the 50 error measures are computed. The statistical analysis aims to assess the variability of the model when several simulations are carried out.

**Table 7:** Statistical results over 50 runs for horizon 15.

Statistic	RMSE	MAE	MAPE	MSLE
Mean	1.32$\times 10^{2}$	1.11$\times 10^{2}$	3.59$\times 10^{-2}$	1.94$\times 10^{-3}$
Std	2.90$\times 10^{1}$	2.48$\times 10^{1}$	8.16$\times 10^{-3}$	8.17$\times 10^{-4}$
Min	9.26$\times 10^{1}$	7.19$\times 10^{1}$	2.37$\times 10^{-2}$	8.21$\times 10^{-4}$
Max	1.88$\times 10^{2}$	1.47$\times 10^{2}$	4.94$\times 10^{-2}$	3.54$\times 10^{-3}$
Median	1.34$\times 10^{2}$	1.07$\times 10^{2}$	3.57$\times 10^{-2}$	2.05$\times 10^{-3}$
Q1 (25%)	1.06$\times 10^{2}$	9.20$\times 10^{1}$	2.92$\times 10^{-2}$	1.23$\times 10^{-3}$
Q3 (75%)	1.55$\times 10^{2}$	1.34$\times 10^{2}$	4.35$\times 10^{-2}$	2.50$\times 10^{-3}$
Range	9.54$\times 10^{1}$	7.56$\times 10^{1}$	2.57$\times 10^{-2}$	2.72$\times 10^{-3}$
IQR	4.84$\times 10^{1}$	4.21$\times 10^{1}$	1.43$\times 10^{-2}$	1.27$\times 10^{-3}$
Skewness	2.48$\times 10^{-1}$	9.59$\times 10^{-2}$	1.96$\times 10^{-1}$	2.86$\times 10^{-1}$
Kurtosis	−1.09	−1.31	−1.24	−9.33$\times 10^{-1}$

The results indicate that the proposed model exhibits stable performance across different random weight initializations. The mean RMSE is $1.32\times 10^{2}$ with a standard deviation of $2.90\times 10^{1}$, while the mean MAE is $1.11\times 10^{2}$ with a standard deviation of $2.48\times 10^{1}$. Likewise, MAPE and MSLE present mean values of $3.59\times 10^{-2}$ and $1.94\times 10^{-3}$, respectively, with low variability across the 50 runs. The proximity between the mean and median for all metrics indicates approximately symmetric distributions, which is consistent with the low positive skewness values ranging from $9.59\times 10^{-2}$ to $2.86\times 10^{-1}$.

Furthermore, the negative kurtosis values observed for all error metrics indicate flatter-than-normal distributions, suggesting that extreme error values occur infrequently. The interquartile ranges are also moderate relative to the corresponding ranges, showing that the central 50% of the runs are concentrated within a limited interval. These statistical results demonstrate that the proposed model provides consistent forecasting performance and is robust to variations caused by random weight initialization.

To further assess the statistical behavior of the prediction errors across the 50 independent runs, the Shapiro-Wilk normality test was applied to the RMSE, MAE, MAPE, and MSLE distributions. The results yielded test statistics of *W=0.93* (*p=0.43*) for RMSE, *W=0.94* (*p=0.47*) for MAE, *W=0.95* (*p=0.59*) for MAPE, and *W=0.95* (*p=0.65*) for MSLE. Since all *p*-values are greater than the significance level of 0.05, the null hypothesis of normality cannot be rejected for any of the evaluated metrics.

These results indicate that the error distributions are consistent with a normal distribution, suggesting that the variability observed across independent runs is primarily attributable to the stochastic initialization of the model rather than to systematic outliers or highly skewed behavior. Furthermore, the approximate normality of the error distributions supports the use of parametric statistical analyses for subsequent comparisons of model performance.

A one-sample Student’s *t*-test was performed to evaluate whether the mean error differed significantly from the reference value. The test yielded a statistic of *t=-133.47* with a corresponding *p*-value smaller than 0.001. Since the *p*-value is well below the significance level of 0.05, the null hypothesis is rejected, indicating that the mean error differs significantly from the reference value. The large absolute value of the *t*-statistic provides strong evidence against the null hypothesis, demonstrating that the observed mean is not attributable to random sampling variation.

### Explainable results

**Fig. 6:**
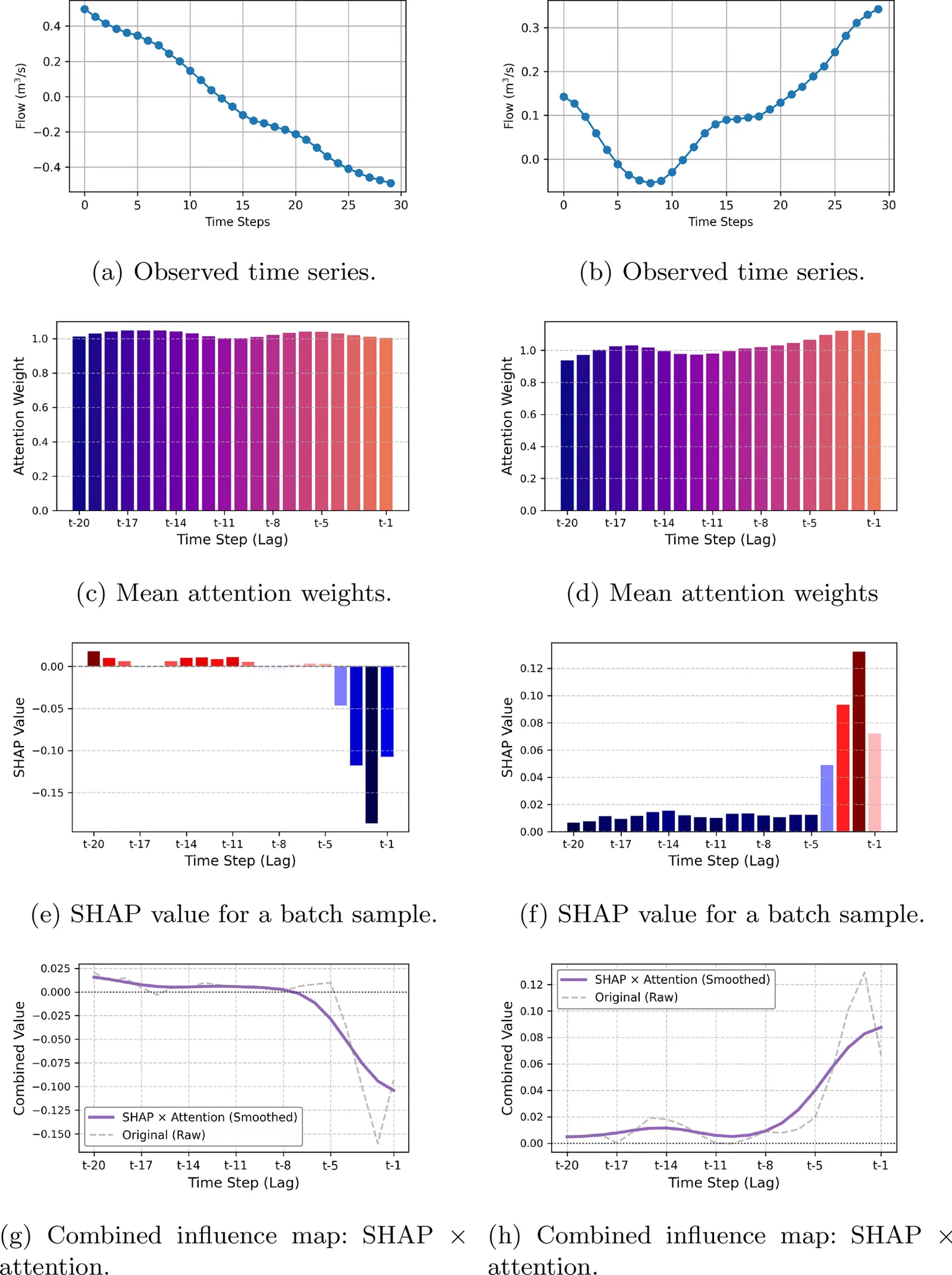
Explainable results for a given time series.

This subsection presents the effect of using explainable methods in our model. Figure 6 (a) and (b) show the observed time series, Fig. 6 (c) and (d) show the MHAW, Fig. 6 (e) and (f) show the SHAP values, and the Fig. 6 (g) and (h) show the combined SHAP *×* attention values.

The weights reflect the distribution of attention across the input window during prediction. For the attention and SHAP values, those values that are far away from the current sample and near the edge of the window (e.g., t-29 in a window size of 30) show erratic values because of the influence from the border values. Due to this, we do not consider these last values.

In Fig. 6 (c) and (d) it is observed the influence of seasonal variability of the time series on the attention weights. Figure 6 (e) and (f) present the SHAP values assigned to each time lag. Positive values (in red) indicate a contribution to increase the forecast, while negative values (in blue) indicate a contribution to decrease it. Quantitative analysis reveals that the majority of the explanatory impact is concentrated in the more recent values of the input window (e.g., $t\text {-}10$ to $t\text {-}1$), which accounts for approximately 85.37% of the total SHAP magnitude.

Figure 6 (g) and (h) present the combined influence map, obtained by computing the element-wise product between SHAP values and attention weights for each time lag. The purple line represents the smoothed influence signal after applying a one-dimensional Gaussian filter, while the gray dashed line corresponds to the raw product. The smoothed curve improves interpretability by reducing local noise and emphasizing overall trends. This combination highlights the time steps that are both attended to by the model and have a meaningful influence on the final prediction.

The plot reveals that the most influential regions are concentrated near the end of the input window, particularly between $t\text {-}6$ and $t\text {-}1$, where the influence varies sharply. This aligns with the expectation that recent lags are often more informative in time series forecasting. However, the curve also shows moderate combined relevance in earlier parts of the window, reflecting the presence of temporal dependencies beyond the immediate past.

The use of SHAP-MHAW thus provides a more reliable and interpretable measure of temporal influence than either method alone where SHAP values quantify the actual contribution of each time step to the model’s prediction, indicating how each lag increases or decreases the forecast, while attention weights reflect where the model is focusing during the decision process, regardless of whether that focus has a positive, negative, or neutral impact.

## Conclusion

This study introduces CNN-Transformer-SHAP-MHAW, a hybrid deep neural network architecture that integrates convolutions and a multi-head attention mechanism to enhance multivariate time series forecasting. By using the pattern recognition of CNNs alongside the dynamic attention mechanisms of transformers, the proposed model can effectively capture both localized temporal features and long-range dependencies. The convolutional component reduces input noise and dimensionality, and extracts high-level features that enhance the representation power of the subsequent transformer layers. The transformer-based backbone applies multi-head attention and adaptive gating mechanisms to emphasize relevant covariates and temporally important patterns, enabling more accurate forecasting.

Experiments were conducted considering a hydroelectric natural flow time series data, demonstrating the superior performance of CNN-Transformer-SHAP-MHAW when compared to several state-of-the-art DL models. The model achieves an MAPE as low as 2.2%, underscoring its effectiveness and robustness across different time series forecasting scenarios. These findings validate the hybrid model’s ability to generalize across domains with varying temporal structures and feature complexities.

By merging attention weights (model focus) and SHAP values (contribution), the resulting influence map improved interpretability, allowing for a more trustworthy understanding of which past inputs drive the forecast. The integration of SHAP values with multi-head attention visualization provides more interpretable results to assist the decision-making process.

The implications of this research extend to numerous real-world applications that demand high-fidelity temporal predictions. The CNN-Transformer-SHAP-MHAW model facilitates the deployment of more accurate and interpretable models in decision-making processes. Future work may explore the integration of external knowledge sources, enhanced interpretability modules, and real-time deployment frameworks to further extend the utility and adaptability of the CNN-Transformer-SHAP-MHAW architecture.

## Data Availability

For future comparisons, the proposed CNN-Transformer-SHAP-MHAW is available for future analysis at: https://github.com/SFStefenon/CNN-Transformer-SHAP-MHAW.
